# The Effects of School-Based Condom Availability Programs (CAPs) on Condom Acquisition, Use and Sexual Behavior: A Systematic Review

**DOI:** 10.1007/s10461-017-1787-5

**Published:** 2017-06-17

**Authors:** Timothy Wang, Mark Lurie, Darshini Govindasamy, Catherine Mathews

**Affiliations:** 1 Health Policy Research Department, The Fenway Institute, Fenway Health, Boston, MA USA; 2 Department of Epidemiology, Brown University School of Public Health, Providence, RI USA; 3 Health Systems Research Unit, South African Medical Research Council, Francie Van Zijl Drive, Parow Valley, Cape Town, PO BOX 19070, Tygerberg, Western Cape South Africa; 4 Adolescent Health Research Unit, Department of Child and Adolescent Psychiatry, University of Cape Town, Cape Town, Western Cape South Africa

**Keywords:** School health, Condom use, Sexual behavior, Adolescent

## Abstract

We conducted a systematic review to assess the impact of school-based condom availability programs (CAPs) on condom acquisition, use and sexual behavior. We searched PubMed to identify English-language studies evaluating school-based CAPs that reported process (i.e. number of condoms distributed or used) and sexual behavior measures. We identified nine studies that met our inclusion criteria, with the majority conducted in the United States of America. We judged most studies to have medium risk of bias. Most studies showed that school-based CAPs increased the odds of students obtaining condoms (odds ratios (ORs) for individual studies ranged between 1.81 and 20.28), and reporting condom use (OR 1.36–3.2). Three studies showed that school-based CAPs positively influenced sexual behavior, while no studies reported increase in sexual activity. Findings suggest that school-based CAPs may be an effective strategy for improving condom coverage and promoting positive sexual behaviors.

## Introduction

Globally, adolescents and young adults (15–24 years) account for approximately 60% of incident sexually transmitted infections (STIs) [1]. According to recent UNAIDS estimates, in 2015 female and males aged 15–24 years accounted for approximately 20 and 14% of new HIV-infections among adults (>15 years), respectively [2]. Annually, an estimated 16 million adolescents (15–19 years) give birth in low- and middle-income countries, with complications from childbirth being the leading cause of mortality among adolescent females [3]. Several key studies and reports have highlighted the limited access that adolescents have to basic sexual reproductive health services (i.e. STIs and pregnancy prevention services) in high-, middle- and low- income countries [4–6]. Due to the burden of these sexual and reproductive health conditions (i.e. HIV and STI acquisition, pregnancy complications) among this population, there is a need to implement strategies that can increase access to and utilization of STI and pregnancy prevention methods. Given that approximately 75% of individuals in the school-going age for secondary education globally are enrolled in secondary school [7], schools may serve as an ideal platform to extend coverage for these services.

The Health Promoting Schools (HPS) concept was initiated by the World Health Organization in the 1980s, and has been adopted by the European and Australian HPS networks [8]. The HPS approach is characterized by a formal health curriculum aimed at providing students with the skills and knowledge needed to make healthy choices, promote a healthy physical and social school environment, and facilitate interaction between communities and schools to promote health [8]. The Comprehensive School Health Program (CSHP) was also developed during this period and was adopted mainly by the United States of America (USA) and Canada [8]. The CSHP includes eight components: sequential health education from grades 1–12, school-based health services, healthy school environments, physical education in schools, food services, counseling services, health promotion among school staff, and school or community integration for health promotion.

While the HPS and CSHP concepts exist, literature suggests that there is a considerable gap between the conceptualization and the implementation in schools. According to a school health census report conducted in the USA between 2010 and 2011, few schools have implemented all of the HPS or CSHP concepts, and few evaluations have been conducted on its implementation [9]. However, in recent years, one component of the CSHP (i.e. school-based health services) has been adopted and evaluated in several schools in the USA. Schools adopting this approach in this setting usually have a school-based health center (SBHC) on the school premises to provide health services that are integrated into school programs [9].

Most of the studies on SBHCs are limited to high-income settings [10]. Studies have shown that SBHCs serve as an effective platform for reducing the structural barriers to accessing care [10]. SBHCs are often operated by nurses, physicians and school staff and seek to provide comprehensive services, including vaccinations, drug and substance abuse counseling, anti-violence and anti-bullying programs, and healthy eating and fitness programs for students [9]. Importantly, these SBHCs provide a range of reproductive health services, with the majority providing services such as STI diagnosis and treatment and pregnancy screening [9]. However, the majority of SBHCs in high-income countries do not distribute contraceptives (i.e. condoms, birth control). According to a USA school census report, 82.1% of SBHCs promote abstinence and 49.8% of SBHCs are actually prohibited from providing contraceptives [9].

Given that contraception is an effective and low-cost method for preventing STIs and pregnancy, lack of availability of contraception is a missed opportunity for SBHCs to help prevent STIs and unwanted pregnancies in adolescents and young adults [4, 10]. However, the minority of SBHCs, primarily in the USA, have started condom availability programs (CAPs) for students. These programs have been controversial, as proponents argue that school-based CAPs could assist in increasing condom use among adolescents, while opponents argue that school-based CAPs could increase sexual activity among adolescents [11]. Due to the controversial nature, few SBHCs currently operate CAPs to distribute condoms to students. To date, no study has systematically reviewed the efficacy of school-based CAPs. A better understanding of the impact of school-based CAPs on students’ sexual behavior could assist program planners and policy makers in their decision-making process around what sexual reproductive health services SBHCs should offer. The objectives of this systematic review were to determine the impact of school-based CAPs on condom acquisition, condom use and sexual behavior outcomes, and to assess the factors that facilitate or impede the delivery of these programs.

## Methods

### Search Strategy

**Fig. 1:**
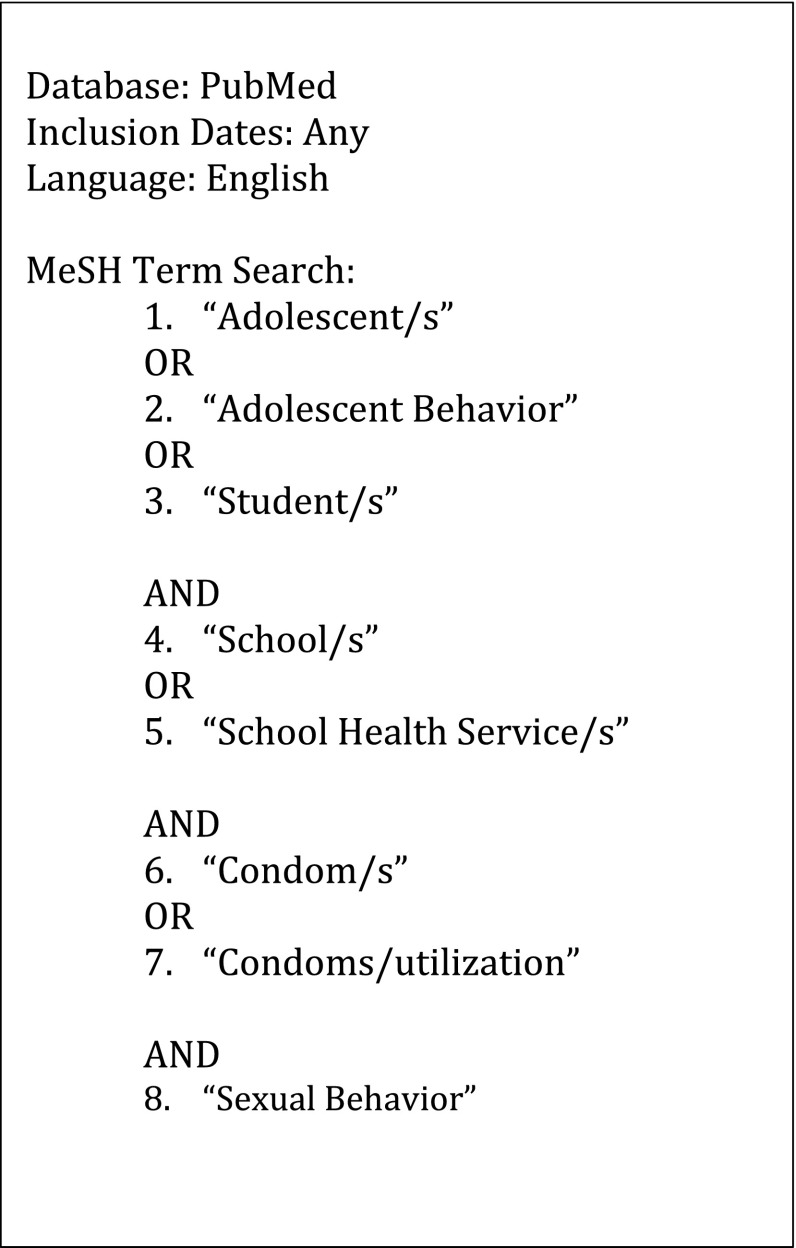
Search strategy

An electronic search was conducted in PubMed using a comprehensive search strategy (Fig. 1) to identify studies assessing school-based programs that made condoms available to students. The search was limited to English language papers, published before February 2016, with no restriction on geographic region. The search strategy included both PubMed’s Medical Subject Headings (MeSH) terms (e.g. school health services, condoms) and sub-terms (e.g. adolescents, sexual behavior, condom utilization). Related citation searches on PubMed were conducted to identify any study that met the inclusion criteria but used less common MeSH terms that were not used in the original search. Additional online databases were searched (i.e. Cochrane library and the Education Resource Information Centre (ERIC)) to identify further articles. Furthermore, co-authors (CM, ML) were contacted for additional citations.

### Inclusion Criteria

In order to be included in this review studies had to evaluate an intervention or program that distributed condoms directly on school premises. In addition, studies had to include process outcomes (i.e. condom acquisition or use by adolescents) and any sexual behavior measures (e.g. sexual activity, age of sexual debut, condom use during sexual intercourse, number of sexual partners), as defined by each study, to be eligible.

### Screening and Data Extraction

Title and abstracts of all citations obtained from the search were screened. Full-text of all potentially eligible studies were retrieved and assessed using the full inclusion criteria. TW and ML assessed whether potentially eligible studies met the inclusion criteria. Included studies were reviewed and all relevant variables were extracted.

### Analysis

**Fig. 2:**
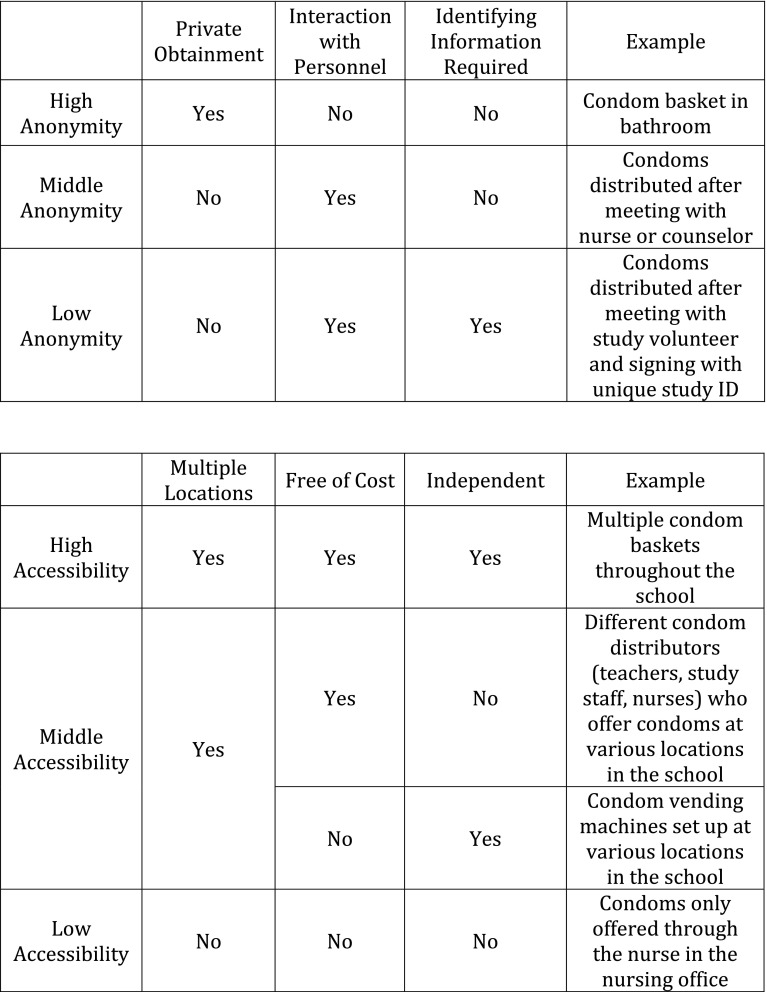
Anonymity and Accessibility Classification Descriptions

A descriptive analysis of key outcome measures was conducted on all included studies. Point estimates or measures of association together with corresponding 95% confidence intervals (CIs) and test statistics were presented for each outcome measure. Preliminary patterns between types of school-based CAP components and program success were assessed based on two criteria (i.e. anonymity and accessibility for the students who wished to obtain condoms). These factors were chosen as internationally these are regarded as key attributes of quality adolescent health services. [12] These programs were ranked (high, middle, low) in the extent to which they maintained anonymity and promoted accessibility (Fig. 2). Anonymity was assessed based on level of privacy when obtaining condoms. The requirement of parental consent was also assessed. Accessibility was based on the ability of a student to independently access condoms at various locations at no cost, without the assistance of a nurse or faculty member.

## Risk of Bias Assessment

We adapted the Effective Public Health Practice Project’s Quality Assessment Tool for Quantitative Studies to assess the risk of bias in each of the included studies [13]. Studies were evaluated for selection, reporting and misclassification bias by assessing and scoring the following factors: study design, confounders, and assessment of exposure and outcome measures. Total study risk of bias was classified accordingly: high (score = 3), medium (score = 2) and low (score = 1).

## Results

### Search Results

**Fig. 3:**
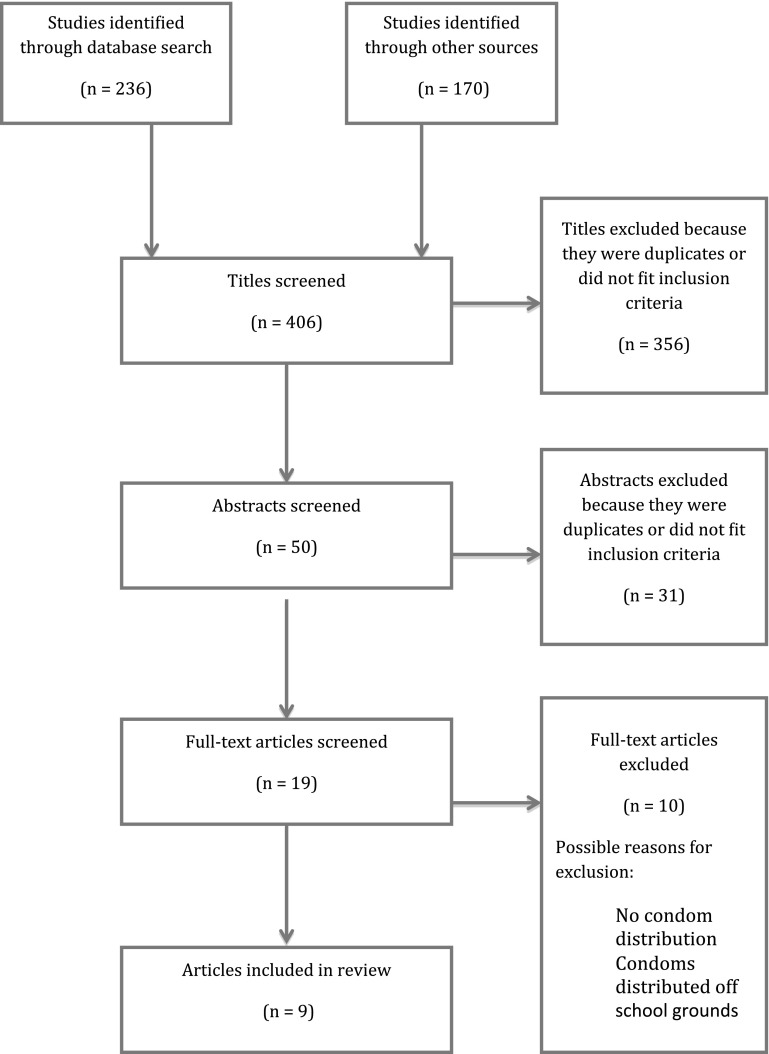
Selection process for the inclusion of studies

The PubMed database search identified 236 potential citations. An additional 170 possible citations were identified through related citation searches and citations from co-authors. Of the 406 combined citations, 356 citations were excluded as they were either duplicates or did not meet the inclusion criteria based on the title screen. The title and abstracts of the 50 remaining potential citations were then examined for inclusion. Overall, 31 studies did not meet the inclusion criteria based on their abstracts; hence the remaining 19 full text articles were retrieved and then assessed for full inclusion. Of the 19 potentially eligible studies, 10 did not meet the full inclusion criteria and were excluded. The remaining 9 studies met the inclusion criteria and were included in this review (Fig. 3). The search was updated in March 2017 to identify new studies and to include both USA and UK English spelling of key terms (behavior/behavior, utilisation/utilization), but no new studies were identified.

### Study Setting and Demographics of Participants

Of the nine included studies, eight were conducted in the USA and evaluated CAPs based in schools in urban areas within Los Angeles, Seattle, Philadelphia, and New York [14–20] (Table 1). The remaining study was conducted in urban schools in Tijuana, Mexico [21]. All nine studies were conducted in public secondary schools with students in the 13–18 years old age range. All the USA-based studies had approximately equal distributions of male and female students; in the Mexico study, approximately 60% of the student population were female [21].

**Table 1:** Condom and sexual behavior outcomes of school-based CAPs

First author, year, country, study design	Sample and participants	Condom acquisition	Condom use	Sexual behavior	Program components
Guttmacher, 1997, USA, Quasi-experimental [11]	7119 students in 12 randomly selected New York schools with condom distribution programs and 5738 students in 10 Chicago schools completed the cross-sectional survey, the schools all had similar gender and racial distribution	Not assessed	Used condom at last sexual intercourse: OR 1.36 (p < 0.01) OR 1.29 (p < 0.01)^a^ OR 1.42 (p < 0.01)^b^ Used condom at last intercourse for students with 3+ partners in past 6 months: OR 1.85 (p < 0.01)	Percentage sexually active: New York: 57.2% Chicago: 57.1% (NS)^a^ New York: 39.6% Chicago: 37.6% (NS)^b^	Anonymity: Low, students receive condoms from clinic volunteers and must leave ID numbers Accessibility: Low, students can’t access condoms independently, only 1 location to receive condoms Consent: Required
Furstenberg, 1997, USA, Longitudinal (with Cohort element) [14]	In 1992, 9 Philadelphia schools implemented health resource centers that distributed condoms, survey data was taken in 1991 and 1993 by interviewing eligible 14–18 year olds in randomly selected census blocks around HRC and non-HRC schools, 490 students responded in 1991, 945 students responded in 1993	Not assessed	Used condom at last sexual intercourse: HRC (all): 52.2–58% (NS) HRC (low): 57–61% (NS) HRC (high): 37–50% (NS) Non-HRC: 61.9–64.6% (NS)	Ever had sex: HRC (all): 64–57.6% (NS) HRC (low): 61–56% (NS) HRC (high): 75–66% (NS) Non-HRC: 55.7–58.8% (NS) Had sex in last 4 weeks: HRC (all): 32–28.6% (NS) HRC (low): no change HRC (high): 43–29% (NS) Non-HRC: 24–25.6% (NS) Had unprotected sex in last 4 weeks: HRC (all): 7.5–5.6% (NS) HRC (low): no change HRC (high): 14–6% (p = 0.08) Non-HRC: 4.8–5.4% (NS)	Anonymity: Middle, students generally must interact with HRC staff to take condoms Accessibility: Low, the majority of schools don’t allow students to access condoms independently, and usually must be accessed through the HRC only Consent: Not required
Schuster, 1998, USA, Longitudinal [15]	1945 students grade 9–12 in a Los Angeles high school took a baseline survey before start of Condom Accessibility Program. 1110 students took the follow-up survey one year later, 52% male, 48% female	Not assessed	Every time: 37–50% (p = 0.005)^a^ 27–32% (NS)^b^ At recent first intercourse: 65–80% (p = 0.038)^a^ Anticipated condom use for sexually inexperienced: 62–90% (p < 0.001)^a^ 73–94% (p < 0.001)^b^	Ever had vaginal intercourse: 55.8–55% (NS)^a^ 45.4–46.1% (NS)^b^ Vaginal intercourse in the last year: 50.6–51.8% (NS)^a^ 42–44% (NS)^b^ Number of times having sex in the past year: 10.6–10.4 (NS)^a^ 11.6–12 (NS)^b^ Percentage with 3 or more sexual partners: 51–48% (NS)^a^ 38–35% (NS)^b^	Anonymity: High, students did not have to interact with any staff or leave any identifying information Accessibility: High, condoms were placed in 4 baskets that students could access easily, independently, and free of cost Consent: Not Required
De Rosa 2012, USA, Quasi-experimental [16]	12 urban California high schools with CAPs were divided into control and intervention groups, with the intervention schools receiving a process evaluation and structural intervention to improve the implementation of the CAPs	Acquired condoms from CAP: OR 1.81 (95% CI 1.32, 2.49) Acquired condoms from CAP among sexually active: OR 3.08 (95% CI 1.77, 5.36)	Not assessed	Ever had sexual intercourse: Intervention: 47.8% Control: 46.1% (p = 0.2) Had sexual intercourse in the past 3 months: Intervention: 77% Control: 77% (p = 0.98)	Anonymity, accessibility, and parental consent of the individual CAPs was not discussed in this study because the study was focused on the components of the structural intervention they were implementing to improve already existing CAPs
Blake, 2003, USA, Cross-sectional [17]	Multistage clustering design to get representative sample of Massachusetts adolescents in high school, high schools with CAP compared to high schools without, 5370 students selected, 4166 completed survey, 50.7% male, 49.3% female	Perceive condoms as easy to acquire: OR 1.3 (95% CI 0.8, 2.1)	Used condom during most recent sex: OR 2.1 (95% CI 1.5, 2.9; p = 0.0001) Used condoms to prevent pregnancy: OR 2.1 (95% CI 1.5, 2.8; p = 0.0001)	Ever had sexual intercourse: OR 0.8 (95% CI 0.6, 0.9) p = 0.0037 Mean age at first intercourse: 14.4 compared to 14.3 (NS) Mean number of partners: 2.8 compared to 2.8 (NS) Had sexual intercourse in the past 3 months: OR 0.8 (95% CI 0.6, 0.9) p = 0.0252	Anonymity: Middle, the majority of the high schools in this study distributed condoms through school nurses or other faculty personnel Accessibility: Low/Middle, the majority of schools did not allow students to access condoms independently, but 10% did use methods like vending machines that allowed students independent access, but not free of cost Consent: Not Required
Kirby, 1999, USA, Quasi-experimental [18]	Pretest survey administered in 10 Seattle high schools before implementation of condom distribution programs (n = 7179), post-test survey administered 2 years later during implementation (n = 7893), results of surveys compared to national survey of high schools (YRBSS) in the same years (schools in the national survey with condom distribution were excluded)	Total number of condoms obtained: 133,711 Condoms obtained per student: 4.4 (’93–’94) 4.7 (’94–’95) Condoms obtained from baskets vs. vending machines: 131,185 from baskets vs. 2526 from vending machines	Used condom at last sex in past 3 months: Seattle: 57–51% YRBSS: 53–56% (p = 0.042)	Ever had sexual intercourse: Seattle: 46–42% YRBSS: 49–50% (p = 0.126) Had sex in the last 3 months: Seattle: 32–28% YRBSS: 35–36% (p = 0.024) 4+ partners lifetime: Seattle: 15–13% YRBSS: 18% to 18% (p = 0.219) 4+ partners in last 3 months: Seattle: 3–2% YRBSS: 3–4% (p = 0.015)	Anonymity: High, all 10 schools distributed condoms through baskets, vending machines, or both so students could access condoms privately Accessibility: Middle/High, vending machines had a cost and baskets were free, both methods allowed students to access condoms independently in multiple locations Consent: Not Required
Ethier, 2011, USA, Cross-sectional [19]	12 urban California high schools, half with school-based health centers, half without, 44% of students indicated that they had ever had sex and were included in analyses, 1226 males, 1374 females	Not assessed	Used condom during last intercourse: CAP: 74.3% No CAP: 71.1% (p = 0.23)^a^ CAP: 59.6% No CAP: 63.4% (p = 0.16)^b^	Because the analytic sample only contained sexually experienced students, this was not an outcome that was assessed	Anonymity: Middle, students had to interact with health services personnel but no identifying information was required Accessibility: Low, student can’t access condoms independently, only 1 location to receive condoms Consent: Required
Wolk, 1995, USA, Cross-sectional [20]	Adams City High School in Colorado, 1200 students in grade 9–12, 152 students randomly sampled, 71 male, 78 female	Not assessed	Benefit-Risk Analysis: the odds of encouraging a sexually active student to use a condom are 3.2 (95% CI 2.1, 4.9) times greater than the odds of encouraging a non-sexually active student to become sexually active	Prevalence of sexual activity: CAP: 59.8% Colorado Estimate: 54.5% (p > 0.05)	Anonymity: Middle, students had to interact with health center staff or faculty reps to get condoms Accessibility: Low, students can’t access condoms independently, only 1 location to receive condoms Consent: Not Required
Martinez-Donate, 2004, Mexico, Quasi-experimental [21]	4 urban schools in Tijuana were randomized, half received HIV prevention workshops and half did not. CAPs were then started in one workshop school and one non-workshop school to create 4 unique conditions: workshop+CAP, CAP only, workshop only, and neither (control). 320 students in total took part in the study, 37% male, 63% female.	Acquired condoms in last 3 months: Workshop+CAP OR 20.28 (p < 0.001) Perceived difficulty to obtain condoms: Workshop+CAP B = −0.78 (p = 0.054)	Not assessed	Initiation of sexual practices: Workshop+CAP HR 0.14 (p < 0.001) Workshop: HR 0.14 (p < 0.001) CAP HR 0.12 (p < 0.001) Sexual intercourse in last 3 months: Workshop+CAP OR 0.54 (p = 0.47) Workshop: OR 0.30 (p = 0.113) CAP OR 0.98 (p = 0.985) Unprotected sexual intercourse in last 3 months: Workshop+CAP OR 0.38 (p = 0.347) Workshop: OR 0.73 (p = 0.73) CAP OR 1.63 (p = 0.678)	Anonymity: Low, students must interact with study staff to get condoms and must use a personal ID card Accessibility: Low, students can’t access condoms independently, condoms only available at 1 location Consent: Not specified

^a^Males only

^b^Females only

### Study Design Characteristics

The majority of studies (8/9) were outcome evaluations of school-based CAPs. Three studies used cross-sectional study designs to compare schools with CAPs to those without CAPs [17, 19, 20]. Four studies used quasi-experimental study designs [16]. Two studies used a longitudinal design with pre- and post-CAP implementation surveys [14, 15]. Studies with longitudinal elements had follow-up times that varied from 6 months to 5 years in length. Outcomes in all studies were ascertained using self-reported surveys.

### Study Results

#### Condom Acquisition

Four studies examined the effects of school-based CAPs on condom acquisition by adolescent participants, and all studies reported an increase in condom acquisition in schools with a CAP [16–18, 21]. The largest effect was observed in a quasi-experimental study in Mexico which evaluated a school-based CAP delivered with HIV prevention workshops [21]. This study found that the odds of acquiring condoms among students in the intervention schools were 20 times more than students in the control schools (odd ratio (OR) 20.28, p < 0.001), albeit the sample size was small (n = 320) [21]. The smallest effect size was observed in a quasi-experimental study in the USA in which the odds of acquiring condoms among students was 1.8 times higher (OR 1.81, 95% CI 1.32–2.49) in intervention schools where CAP implementation was strengthened [16]. Furthermore, this study found that the odds of acquiring condoms among sexually active students in the intervention group were three times more than the control group (OR 3.08, 95% CI 1.77–5.36) [16].

#### Condom Use

Seven studies examined the effects of school-based CAPs on condom use. Two observational studies from the USA showed no significant differences in condom use [14, 19], whereas one school survey showed a 6% decrease in condom use in last 3 months in schools with CAPs (57–51% p = 0.042) [15]. The authors of this study hypothesize that a substitution effect may have been present as students may have substituted condoms accessed from their school for condoms they acquired previously from the community, which may have led to this marginal decrease in condoms use [15]. The remaining three studies showed increases in condom use [11, 17, 20]. The largest effect was observed in a cross-sectional study in the USA which showed that the odds of using condoms (OR 2.1, 95% CI 1.5–2.9) or using condoms as a form of contraception (OR 2.1, 95% CI 1.5–2.8) were two times higher among students that attended schools with CAPs [17]. A similar effect was also observed in a cross-sectional study conducted in the USA (OR 1.36; p < 0.01) [9]. Moreover, this study showed that the odds of using a condom during the last sexual intercourse within the past 6 months among sexually active students from schools with CAPs almost doubled (OR 1.85, p < 0.01) [11].

#### Sexual Behavior

Eight studies assessed the impact of school-based CAPs on sexual behavior. Overall, five studies showed no significant differences in sexual behavior outcomes as measured by exposure to sexual intercourse within the last 3 months, frequency of sexual intercourse, and prevalence of multiple partners [11, 14–16, 20]. No study reported a significant increase in sexual activity among students attending schools with CAPs. However, three studies reported a significant decrease in some of their sexual behavior outcomes because of the CAPs [17, 18, 21]. The largest effect was observed in a quasi- experimental trial conducted in urban schools in Mexico [21]. This study found that students in the intervention group who were exposed to school-based CAPs and HIV prevention workshops had an 86% decreased risk of initiating sexual practices compared with students who attended schools in the control group (HR 0.14, p < 0.001) [21]. It is unclear what factors may have led to this finding in this study as key program attributes (anonymity and accessibility) were judged to be “low” [21] compared to other studies in the USA with programs that we considered to have medium to high anonymity and accessibility.

A cross-sectional study found that the odds of ever having sexual intercourse or having sexual intercourse in the past 3 months were 20% lower among students in schools with CAPs, (OR 0.8, 95% CI 0.6–0.9, p = 0.0037) and (OR 0.8, 95% CI 0.6–0.9, p = 0.0252), respectively [17]. Furthermore, 2 years’ post-implementation of a school-based CAP in public high schools in the USA was associated with a 4% decrease in the percentage of students having sex in the last 3 months (32–28%) [15].

#### School-Based CAP Components

None of the included studies assessed factors facilitating or impeding delivery of the school-based CAP. Overall, four studies were judged to have implemented school-based CAPs with medium levels of anonymity (Table 1). The majority of studies (n = 5) were judged to have implemented school-based CAPs with low accessibility as condoms were not available at multiple locations or could not be accessed independently by the student. Two studies from the USA indicated that parental consent was required for students to access CAPs [14, 19]. There was no distinct pattern identified between condom acquisition or use and the anonymity or accessibility of the CAP or parental consent as almost all studies found that CAPs were positively associated with condom acquisition and/or condom use.

#### Risk of Bias

Five studies were judged to have moderate risk of bias as authors employed rigorous study designs and adjusted for confounders or reported adequate survey completion rates (60–80%) [14–18] but were prone to potential selection bias (Table 2). Three studies were judged to have high risk of bias as there was a lack of information regarding selection of participants and participant completion rate was low [11, 19, 20]. One study was judged to have low risk of bias as the study did not report information regarding the validity or reliability of their measurement tool [21].

**Table 2:** Risk of bias assessment

Author	Selection bias	Study design	Confounders	Data collection method: exposure measure	Data collection method: outcome measure	Global rating	Comments
Guttmacher [11]	2	3	1	3	3	3	Cross-sectional design Validity and reliability for exposure measures not described
Furstenberg [14]	2	2	1	3	3	2	Validity and reliability for exposure and outcome measures not described
Schuster [15]	1	2	1	3	3	2	Validity and reliability for exposure and outcome measures not described
De Rosa [16]	1	2	1	3	3	2	Validity and reliability for exposure and outcome measures not described
Blake [17]	2	3	1	1	3	2	Cross-sectional design Validity and reliability for outcome measure not descried
Kirby [18]	1	2	1	3	3	2	Validity and reliability for exposure and outcome measures not described
Ethier [19]	2	3	2	3	3	3	Cross-sectional design Validity and reliability for exposure and outcome measures not described
Wolk [20]	3	3	2	3	3	3	Percentage of selected students who agreed to participate not described Cross-sectional design. Validity and reliability for exposure measures not described
Martinez-Donate [21]	2	2	1	3	2	1	Validity and reliability for exposure measures not described

## Discussion

The aim of this review was to evaluate the effects of school-based CAPs on condom acquisition or condom use and sexual behavior. Most studies were conducted in urban public high schools in the USA. Overall, results from this review suggest that school-based CAPs are effective in increasing condom acquisition and use, and have a positive influence on adolescent sexual behavior. Findings suggest that school CAPs may be an effective strategy for improving condom coverage and promoting positive sexual behaviors among this vulnerable population.

School-based programs have been shown to improve students’ access to and uptake of health services, as found in an earlier published review [10]. In our review, most studies showed that students in schools with CAPs were more likely to have obtained condoms than students in schools without CAPs. Our review suggests that when school-based CAPs are combined with school-based HIV prevention educational programs this leads to a greater impact on condom acquisition, although this was only investigated in one study. [21].

The odds of students acquiring condoms, particularly among sexually active adolescents, were almost doubled among students attending schools that strengthened the implementation of school-based CAPs through health promotion activities and enhanced co-ordination between the school clinic and campus [16]. Most studies also reported increases in condom use among students who attended schools with CAPs, particularly among sexually active students in their last sexual intercourse [11, 17, 20]. This review did not observe a pattern between significant increase in condom use and the anonymity, accessibility, and consent requirements of the CAP. This suggests that there could be other potential factors which influence student’s decision to obtain and use condoms from school-based CAPs besides anonymity and accessibility (e.g. environmental context), and warrants further investigation. Overall, these findings suggest that integration of school-based CAPs with other school health services may be effective in increasing condom access and utilization, particularly among high-risk groups.

There is a perception that providing condoms at schools may promote negative sexual behaviors and increase sexual activity. However, no study included in this review reported an increase in sexual activity among students at schools with CAPs. Three studies showed that school-based CAPs were associated with positive sexual behavior outcomes [17, 18, 21], and this effect (88% decrease in risk of initiating sexual intercourse) was largest when school-based CAPs were delivered with HIV prevention workshops [21]. Perhaps these workshops reinforced the benefits of condom use and promoted abstinence.

While the sample size of this review was limited, the findings suggest that CAPs can be effective in increasing condom access and utilization without promoting negative sexual behaviors or causing increases in sexual activity. The findings of this review suggest that schools should consider integrating CAPs along with other health services provided to students. The limited number of eligible studies suggests the value of additional research on school-based CAPs and their effects. In particular, future research should explore the comparative effectiveness of different types of school-based CAPs, and investigate the impact of various program attributes such as anonymity and accessibility. To guide program planners and policy makers, we need further research on the cost and cost-effectiveness of different school-based CAPs and the barriers and facilitators of school-based CAP delivery.

Given that our review suggests school-based CAPs are likely to be effective at increasing the acquisition and use of condoms, school-based CAPs should be considered as one part of a comprehensive, multi-component strategy to prevent STIs and unwanted pregnancy among adolescents. Researchers have recently evaluated such a broad, multi-faceted strategy implemented in the United Kingdom, one component of which was policy to support schools to distribute contraception and condoms. [22, 23] The evaluation showed that over 16 years, during which the strategy was implemented, there was a 51% decrease in the under-18 conception rate. [22, 23] The number of schools establishing CAPs was not reported in the evaluation. [22, 23].

The main strengths in this review are the use of a broad search strategy, with no country restriction, and assessment of risk of bias among included studies. Although the search was confined to one electronic database (PubMed), we searched two extra online databases (Cochrane library, ERIC) and were unable to identify any further eligible studies. Weaknesses include that only English manuscripts were eligible for inclusion; the actual number of duplicate articles and studies which were included was not adequately recorded in the initial screening; and only co-authors in this review were contacted for additional articles. Furthermore, this review found that the evidence-base is subject to several limitations. Firstly, urban areas in the USA were disproportionately represented in this review, possibly due to the fact that there might be fewer school-based CAPs operating outside of urban areas in the USA. Secondly, no studies were identified from low- and middle-income countries, and it is unclear whether similar patterns may be observed in these settings. Thirdly, most of the included studies used observational study designs with self-reported outcome measures, with moderate risk of bias; hence, caution should be applied when reviewing the results of those studies.

In conclusion, it appears that school-based CAPs have the potential to have a positive impact on improving sexual health among adolescents who are affected by a disproportionately large proportion of disease burden from STIs and HIV. This review shows that many of the school-based CAPs evaluations have reported statistically significant increases in condom acquisition and condom use among students. Findings suggest that school-based CAPs may be an effective strategy for improving condom coverage, and promoting positive sexual behaviors. However, further research is needed to rigorously assess these associations, particularly research employing experimental study designs, including studies assessing factors that facilitate and impede the implementation of school-based CAPs, condom access and use.
